# Functional Connectivity Differences in the Insular Sub-regions in Migraine without Aura: A Resting-State Functional Magnetic Resonance Imaging Study

**DOI:** 10.3389/fnbeh.2017.00124

**Published:** 2017-06-28

**Authors:** Zhi-bo Yu, Yan-bing Lv, Ling-heng Song, Dai-hong Liu, Xue-ling Huang, Xin-yue Hu, Zhi-wei Zuo, Yao Wang, Qian Yang, Jing Peng, Zhen-hua Zhou, Hai-tao Li

**Affiliations:** ^1^Department of Radiology, The First Affiliated Hospital, Third Military Medical UniversityChongqing, China; ^2^Department of Medical Imaging, PLA No.324 HospitalChongqing, China; ^3^Department of General Surgery, PLA No.324 HospitalChongqing, China; ^4^Department of Nursing, Chongqing Three Gorges Medical CollegeChongqing, China; ^5^Department of Neurology, The First Affiliated Hospital, Third Military Medical UniversityChongqing, China

**Keywords:** migraine without aura (MWoA), insula, pain, brain function, functional connectivity (FC), functional magnetic resonance imaging (fMRI)

## Abstract

**Objective:** The objective of this study was to investigate resting-state functional connectivity (FC) differences in insular sub-regions during the interictal phase in patients with migraine without aura (MWoA).

**Methods:** Forty-nine MWoA patients (MWoA group) and 48 healthy individuals (healthy control group) were recruited for this study. All of the subjects underwent neurological examination and magnetic resonance imaging (MRI). The MRI data were processed using Brat 1.0 software to obtain a whole-brain FC diagram and using Rest 1.8 software to obtain the FC z-score of the sub-regions of both insulas (six sub-regions on each side). Therefore, there were a total of 12 regions of interest (ROIs) that were used as seed points for the statistical analysis.

**Results:** There was abnormal FC between the insular sub-regions and multiple brain regions in the MWoA patients compared with the healthy control group, and a clear laterality was also observed. In addition, the FC z-score of certain sub-regions was negatively correlated with the disease duration.

**Conclusion:** Different insular sub-regions are functionally associated with different regions of the brain and therefore have different functions. In MWoA, the FC between the insular sub-regions and other brain regions was mostly reduced, while a small amount was increased; additionally, the FC may be ipsilateral with a right-side advantage. Variations in the FC of insular sub-regions can be observed as an important indicator of MWoA.

## Introduction

Migraine is a debilitating disorder and one of the most common disorders of the nervous system (Stovner et al., 2007). This disease has a clear familial aggregation (Yu et al., 2012) and seriously affects the daily life and work of patients. Recurrent migraines may increase the risk of subtle lesions in certain brain regions, which can lead to the functional impairment of these regions (Schwedt and Dodick, 2009). However, the pathophysiological and neurological mechanisms of migraines are not clear.

In recent years, damage to brain structures and abnormal functional connectivity (FC) have been observed using a variety of imaging techniques in the insula of migraine patients (Cauda et al., 2011; Lee et al., 2016; Wilcox et al., 2016). However, only the gross anatomy of the brain has been investigated in previous studies (Borsook et al., 2016; Wang et al., 2016). With a deeper understanding of migraine, the accumulated evidence has shown that the functions of different brain sub-regions vary widely, raising new demands, and challenges for future studies.

The insula is located deep in the Sylvian fissure and is concealed by the frontal, parietal, and temporal lobes (Guenot et al., 2004). The insula is considered to be the hub of activity in migraines and participates in many processes, including goal-oriented conscious awareness (Henderson et al., 2008, 2012; Borsook et al., 2016), cognitive function (Bossaerts, 2010; Wiech et al., 2010; Nieuwenhuys, 2012; James et al., 2013), self-regulation (Preuschoff et al., 2008), external sensation (Wright et al., 2004), and internal sensation (Fasold et al., 2002; Napadow et al., 2013). In previous studies, we found that the mean cortical thickness of the insula in patients with migraine without aura (MWoA) was significantly decreased compared with that in a healthy control group, and the mean cortical thickness of the insula anterior was positively correlated with the duration of the disease course, while the mean cortical thickness of the insula superior and insula inferior was negatively correlated with the duration of the disease course (Yu et al., 2016). Compared with healthy control subjects, decreased regional homogeneity values of patients with MWoA were found mainly in the insula (Yu et al., 2013). The human insula is composed of 5–7 gyri and is divided into the anterior and posterior insula by the central sulcus of the insula (Türe et al., 1999). Different regions of the brain are connected with the various insular gyri and exhibit different functions (Cerliani et al., 2012; Almashaikhi et al., 2014). Therefore, we hypothesized that the insula plays an important role in migraine. The mean thickness and regional homogeneity of the insula were altered in MWoA patients. However, is there a difference in the functional connections between the insula and the whole brain? How does the insula regulate the function of the entire brain in MWoA patients? These questions required further study.

In 1985, Mesulam and Mufson proposed that the primate insula could be essentially divided into three zones based on organizational structure, namely, the agranular, dysgranular, and granular zones (Mesulam and Mufson, 1985). A recent study of insular neurons of the primate insula found a gradual change in insular neurons from the granular neocortex of the dorsal posterior insula to the agranular neocortex of the ventral anterior insula (Bauernfeind et al., 2013). According to previous studies, Morel et al. plotted a magnetic resonance spectrum of human insular sub-regions using a high-resolution MRI technique based on microscopic insular anatomy and the cellular structure of postmortem brains (Morel et al., 2013). Inspired by the study of Morel, Fan generated an atlas of human insular sub-regions based on FC (http://atlas.brainnetome.org/download.html). This atlas divided the insula into six sub-regions, with a total of 12 sub-regions in the bilateral insula (Label ID from 163 to 174); detailed information is shown in Table 1 (Fan et al., 2016). Fan also found that the dorsal agranular insula (dIa) was involved in perception, such as in pain somesthesis, actions, such as inhibition and cognition. This insular subregion has been observed to be significantly activated by paradigms associated with pain monitoring/discrimination, reward tasks, and Sternberg tasks. This atlas provides a basis for our studies of insular sub-regions in MWoA.

**Table 1:** The name and coordinates of insula subregions.

**Label ID**.	**Left and right hemispheres**	**Subregion**	**Abbreviations**	**MNI** 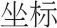		
				***X***	***Y***	***Z***
163	L	The hypergranular insula	L-G	−36	−20	10
164	R	The hypergranular insula	R-G	37	−18	8
165	L	Ventral agranular insula	L-vIA	−32	14	−13
166	R	Ventral agranular insula	R-vIA	33	14	−13
167	L	Dorsal agranular insula	L-dIa	−34	18	1
168	R	Dorsal agranular insula	R-dIa	36	18	1
169	L	Ventral dysgranular and granular insula	L-vId/vIg	−38	−4	−9
170	R	Ventral dysgranular and granular insula	R-vId/vIg	39	−2	−9
171	L	Dorsal granular insula	L-dIg	−38	−8	8
172	R	Dorsal granular insula	R-dIg	39	−7	8
173	L	Dorsal dysgranular insula	L-dId	−38	5	5
174	R	Dorsal dysgranular insula	R-dId	38	5	5

Based on the above research, the insula is a hub of migraine activity; the structural and functional changes of the insular sub-regions are closely related to MWoA. Therefore, the aim of this study was to investigate whether there is a difference in FC between the insular sub-regions and the whole brain in MWoA patients using resting-state functional magnetic resonance imaging (fMRI).

## Materials and methods

### Subjects of the study

#### The MWoA group

Forty-nine patients who were diagnosed with MWoA at the Department of Neurology at the First Affiliated Hospital of the Third Military Medical University from November 2014 to May 2016 were recruited for this study. The inclusion criteria were as follows: (1) the patients met the MWoA criteria of the International Classification of Headache Disorders, 3rd edition, beta version (ICHD-3 beta); (2) the patients had a score of 50 points or more according to the headache impact test-6 (HIT-6); (3) the patients had a score of 4 points or more according to the visual analog scale (VAS) for the assessment of the headache degree; (4) the patients had pain degrees above II according to the World Health Organization (WHO) classification of pain; (5) the patients had no history of drug or preventive medicine abuse; (6) the patients had normal examination results on a conventional MRI scan and neurological examination; (7) the patients had no other mental diseases or comorbidities in addition to migraine; and (8) the patients were free from migraine 3 days before scanning and 4 days after scanning to ensure that they were in the interictal phase. One patient was excluded from the study because of a migraine attack on day 3 after the MRI scan. Ultimately, 48 patients were recruited into the MWoA group (case group), including 11 males and 37 females (all patients were right-handed). The duration time of each migraine attack in all patients was longer than 5 h. The patients had the typical accompanying symptoms and had to rest in a supine position to alleviate the pain.

#### The healthy control group

Forty-eight right-handed healthy volunteers were recruited from the community as the normal control group. This group included 37 females and 11 males. None of the healthy control subjects were immediate family members of the patients with migraine.

#### Clinical assessment scale

General information for the enrolled patients, including the duration of migraine disease and medication history, was recorded. A Neuropsychiatric Inventory (NPI) was conducted to assess the general psychological condition of the subjects. The Hamilton Depression Rating Scale (24-item version, HAMD-24), Hamilton Anxiety Rating Scale (14-item version, HAMA-14), and Chinese Classification of Mental Disorders (3rd edition, CCMD-3) were used to assess the condition of the subjects with respect to depression, anxiety, and bipolar disorder, respectively. The subjects with normal test results were included in the study. For the MWoA group, a short-form headache survey, HIT-6, and the VAS were used to assess the severity of headache and the impact of migraine on the daily activities of the patients.

No statistically significant differences were observed between the two groups with respect to education level, gender, or age (*P* > 0.05). All of the subjects who volunteered to participate in this study were informed of the experimental methods, purpose, and possible risks and discomforts. All of the patients signed the informed consent forms. The study was approved by the Ethics Committee at the First Affiliated Hospital of the Third Military Medical University.

### Data collection

The MRI data were collected using a Siemens 3.0 T MR Scanner (Germany) with an 8-channel head coil. The subjects' heads were stabilized to keep them stationary. First, conventional T1-weighted imaging (T1WI) and T2-weighted imaging (T2WI) were performed to exclude organic lesions in the heads of the subjects; these images were collected in the axial plane. The resting-state functional imaging (T2WI) used echo planar imaging (EPI) for a sequence acquisition of 240 time points. The scan parameters were set as follows: TR = 2,000 ms, TE = 30 ms, slice thickness = 3 mm, number of layers = 36, flip angle = 90°, field of view = 192 mm^2^, and a matrix of 64 × 64.

### Data processing

The raw data obtained by MRI were used for format conversion and classification. The resting-state fMRI data were imported into the Brat 1.0 software (https://www.nitrc.org/projects/brat/) for image preprocessing. The main steps included slice timing, realigning, normalization, denoising, filtering, and smoothing. First, corrections were made to account for the time difference between data acquisition at each point in time. Second, the data at alltime points were spatially aligned with the data collected at the first time point to obtain the head motion parameters of the subject in the scanning time series. Third, all the collected data were resampled according to the Montreal Neurological Institute (MNI) standard template space with a 3 ^*^ 3 ^*^ 3 voxel size for spatial normalization. Fourth, signals from white matter, cerebrospinal fluid, and the head as well as other signals were removed, leaving the gray matter signal for denoising. Fifth, a band-pass filter (0.01 Hz < f < 0.08 Hz) was applied to remove physiological and high frequency noise. Lastly, a Gaussian kernel of 4 mm full width at half-maximum (FWHM) was used to smooth the images with the aim of reducing noise and residual differences. After these steps were performed, a whole-brain functional image was obtained in the standard space. Furthermore, a diagram of the whole-brain FC was calculated using Brat software, with the insular sub-regions as the seed points; the insular sub-regions were the hypergranular insula (G), ventral agranular insula (vIa), dorsal agranular insula (dIa), ventral dysgranular and granular insula (vId/vIg), dorsal granular insula (dIg), and dorsal dysgranular insula (dId). Rest V1.8 (http://restfmri.net/forum/index.php?q=rest) was used to extract the z-score of the FC strength, with the insular sub-regions (L/R-G, L/R-vIa, L/R-dIa, L/R-vId/vIg, L/R-dIg, and L/R-dId, a total of 12 sub-regions) as regions of interests (ROIs) for statistical analysis (http://atlas.brainnetome.org/download.html).

### Statistical analysis

The subjects were divided into two groups: the MWoA group and the healthy control group. The statistical analysis was performed using SPSS 22.0 version (http://www.spss.com) and SPM8 (http://www.fil.ion.ucl.ac.uk/spm). The demographic data and clinical variables (except for gender) of the two groups were analyzed using a two-sample *t*-test, and gender was analyzed using a four-fold table chi-square test. Taking insular sub-regions as seed points, the whole-brain FC in the two groups was analyzed using a two-sample *t*-test with SPM8 software (FWE corrected, *P* < 0.05, clusters > 85). Then, using the z-score as the dependent variable and sex, age, and years of education as the covariates, the test level was set to *P* < 0.05 to compare differences in the FC strength of the insular sub-regions between the two groups. The insular sub-regions which was statistically significant regarding the z-score, the correlation between the FC strength and the duration of the disease course were verified by Pearson's test.

## Results

### Clinical data and assessment outcomes

The demographic and general clinical data of all the subjects are shown in Table 2. The MWoA group and the healthy control group both included 48 subjects. No statistically significant differences were present between the groups with respect to gender, age, years of education, HAMD, or HAMA scores (*P* > 0.05). All the subjects had HAMD scores less than eight points and HAMA scores less than seven points. The disease duration of the MWoA patients ranged from 1 year to 24 years. The HIT scores of the MWoA patients were higher than 50 points, and their VAS scores were higher than 4 points.

**Table 2:** Demographic and clinical data.

	**Controls (*n* = 48)**	**Migraineur (*n* = 48)**	***p*-value**
Gender (male/female)	11/37	11/37	1
Age (years)	35.12 ± 9.45(16–50)	35.47 ± 9.91(16–51)	0.683
Education (years)	11.68 ± 4.57(5–19)	11.30 ± 4.33(5–19)	0.595
HAMD-24	4.56 ± 2.62(0–8)	4.64 ± 2.71(0–8)	0.524
HAMA-14	2.61 ± 1.82(0–7)	2.94 ± 2.07(0–7)	0.437
Disease duration(years)	NA	9.38 ± 6.86(1–24)	
HIT-6 scores	NA	66.40 ± 9.39(50–78)	
VAS scores	NA	7.23 ± 1.45(4–10)	

*Data are expressed as the mean ± SD (range). HAMD-Hamilton Depression Rating Scale; HAMA-Hamilton Anxiety Rating Scale; NA = not applicable*.

### FC results

**Figure 1:**
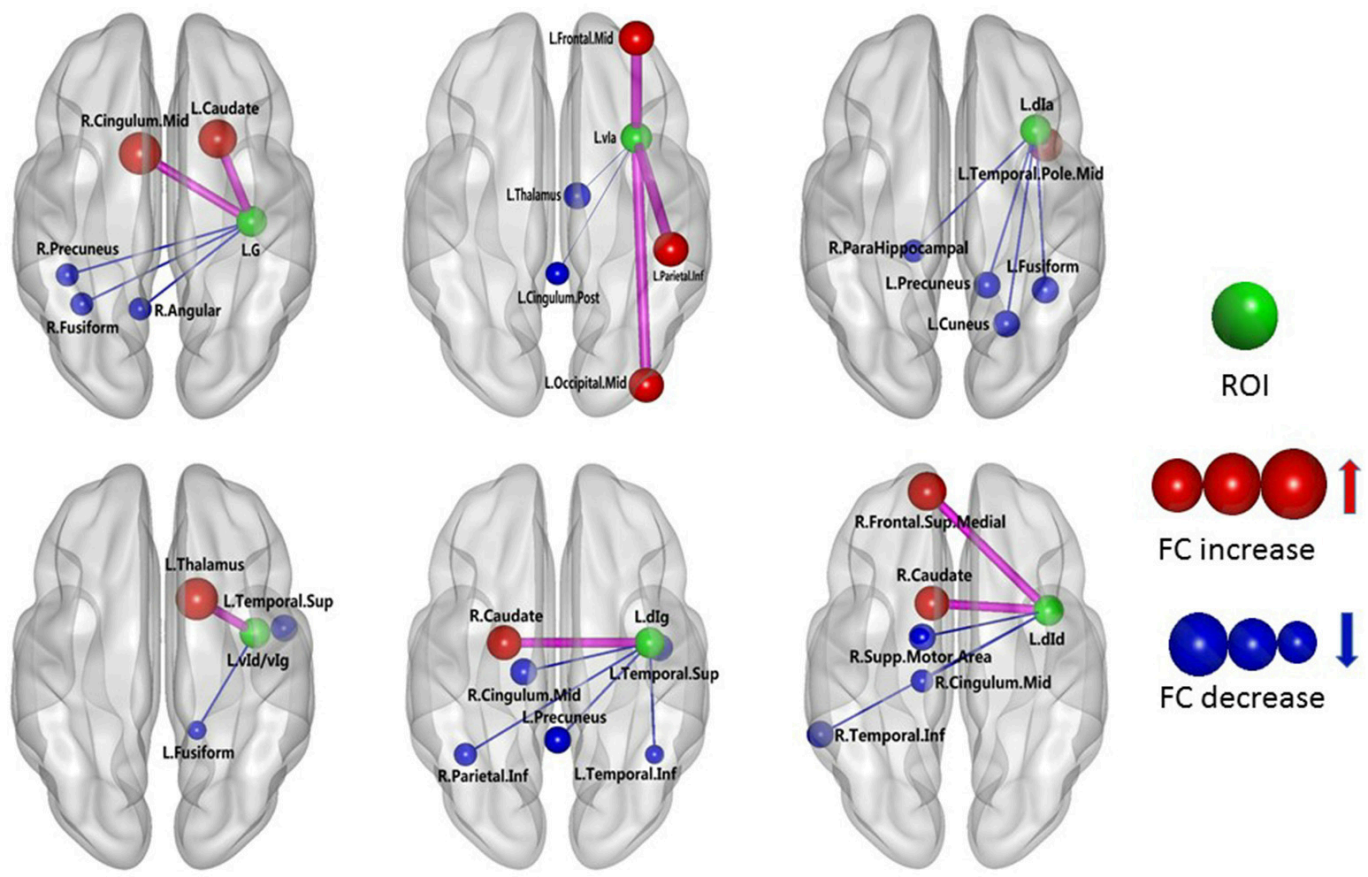
MWoA patients compared with HC group, the difference in the FC between the left insular sub-regions and the whole brain. The green ball represents the seed point; the red ball represents the FC-enhanced brain region; the blue ball represents the FC-reduced brain region; the radius of the sphere represents the size of the functional connection strength; the pink rod represents the FC-enhanced connection; the blue rod represents the FC decrease, and the diameter of the rod indicates the magnitude of the joining strength.

**Figure 2:**
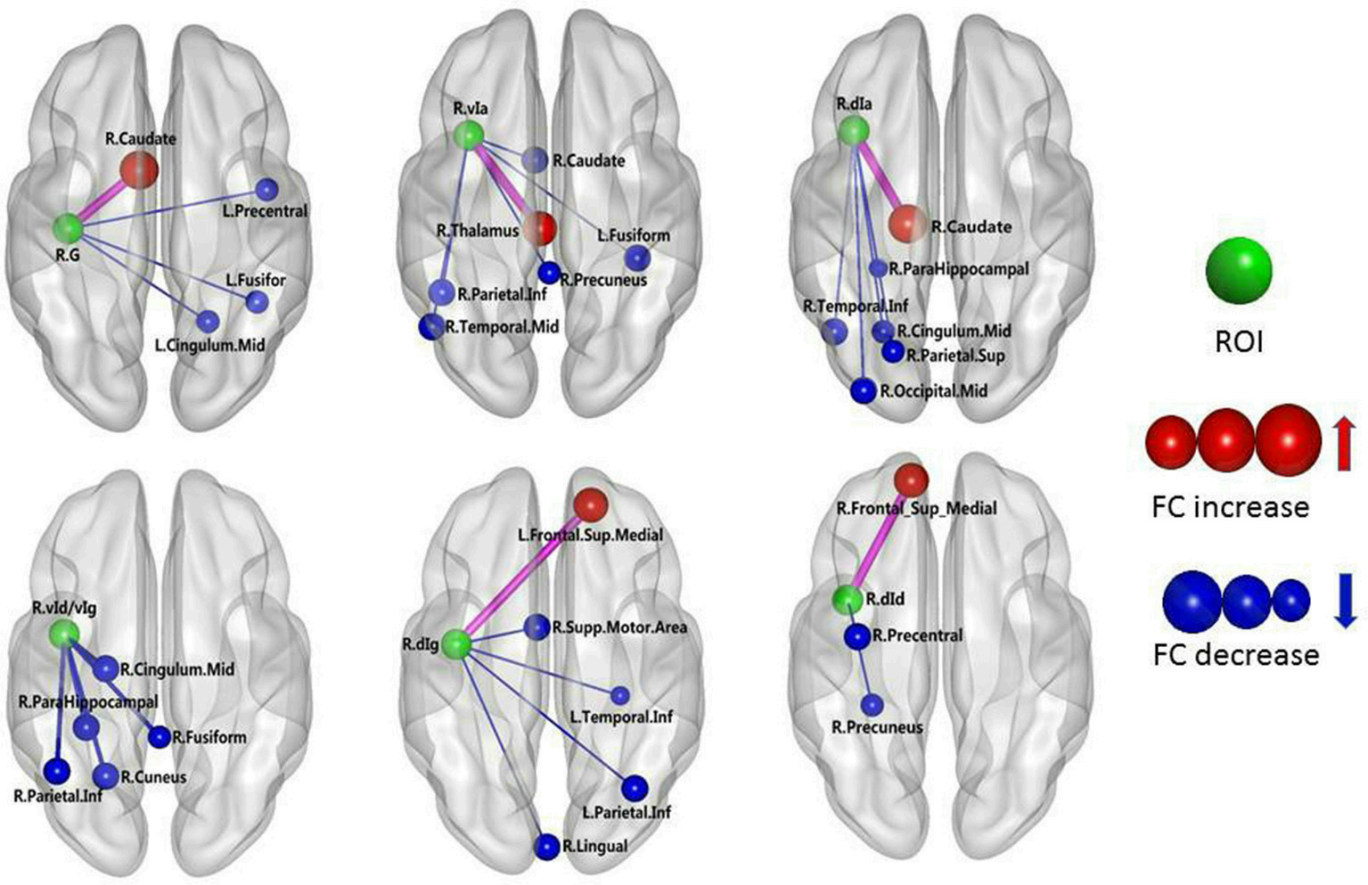
MWoA patients compared with HC group, the difference in the FC between the right insular sub-regions and the whole brain. The green ball represents the seed point; the red ball represents the FC-enhanced brain region; the blue ball represents the FC-reduced brain region; the radius of the sphere represents the size of the functional connection strength; the pink rod represents the FC-enhanced connection; the blue rod represents the FC decrease, and the diameter of the rod indicates the magnitude of the joining strength.

The results of the two-sample *t*-test of FC with insular sub-regions as the seed points for both the MWoA group and the healthy control group are shown in Table 3. There were 58 brain regions in the MWoA group that exhibited significant differences compared with the healthy control group; 15 regions showed enhanced FC (primarily in the frontal lobe, the caudate nucleus, and the thalamus), and 43 regions showed decreased FC (primarily in the temporal lobe, parietal lobe, cingulate gyrus, precuneus, parahippocampal gyrus, and caudate nucleus). The study revealed variation in FC between the right cingulate gyrus/right caudate nucleus and five seed points. Variation in FC was also observed between the left fusiform gyrus and four seed points as well as between the right precuneus/right parahippocampal gyrus/right inferior parietal lobule angular gyrus and three seed points. More detailed information is shown in Figures 1, 2. Notably, the brain regions with FC variations using L/R-vIa, L/R-dIa, L/R-vId/vIg, and R-dId as seed points were located in the ipsilateral brain hemisphere; however, the brain regions with FC variations using the L-dId as a seed point were located at the contralateral brain hemisphere. Of the 58 brain regions with FC variations, more regions were in the right side of the brain (35 regions) than in the left side (23 regions).

**Table 3:** Brain regions with difference of insula subregion functional connectivity in MWoA vs. healthy control.

**ROI**	**Brain region of FC difference**	**Lat**.	***t***	**MNI**			**Cluster**	***p***
				**X**	**Y**	**Z**		
**Insula_G**		**L**						
	Fusiform	R	−3.6336	34	−54	−10	190	0.00028
	Precuneus	R	−3.7322	10	−56	20	302	0.00019
	Angular	R	−3.4240	40	−42	34	44	0.00062
	Cingulum_Mid	R	5.3965	10	8	16	163	0.00001
	Caudate	L	4.0907	−22	14	34	66	0.00004
**Insula_vIa**		**L**						
	Occipital_Mid	L	3.1672	−36	−98	−2	116	0.00154
	Thalamus	L	−3.8223	−8	−10	−12	44	0.00013
	Frontal_Mid	L	3.5541	−32	64	20	72	0.00038
	Cingulum_Post	L	−4.4173	0	−42	24	90	0.00001
	Parietal_Inf	L	3.7365	−46	−32	48	390	0.00019
**Insula_dIa**		**L**						
	Temporal_Pole_Mid	L	3.1869	−38	12	−36	111	0.00144
	ParaHippocampal	R	−4.4610	16	−32	0	100	0.00001
	Fusiform	L	−4.0860	−38	−48	−8	254	0.00004
	Precuneus	L	−3.8755	−14	−46	6	42	0.00011
	Cuneus	L	−3.1881	−22	−62	18	104	0.00143
**Insula_vId/vIg**		**L**						
	Fusiform	L	−5.0383	−14	−44	4	424	0.00001
	Temporal_Sup	L	−3.3344	−50	−2	−8	212	0.00085
	Thalamus	L	5.4447	−14	10	18	93	0.00001
**Insula_dIg**		**L**						
	Temporal_Inf	L	−5.1287	−40	−54	−18	281	0.00001
	Temporal_Sup	L	−3.3585	−42	−10	−18	168	0.00078
	Cingulum_Post	L	−3.4199	0	−48	8	36	0.00063
	Caudate	R	3.9802	22	−8	28	94	0.00007
	Parietal_Inf	R	−4.2341	38	−54	24	297	0.00002
	Cingulum_Mid	R	−3.8487	14	−20	34	248	0.00012
**Insula_dId**		**L**						
	Temporal_Inf	R	−3.6665	56	−46	−16	275	0.00025
	Caudate	R	3.9981	10	8	16	176	0.00006
	Frontal_Sup_Medial	R	4.3521	12	54	16	327	0.00001
	Cingulum_Mid	R	−4.0618	14	−24	32	49	0.00004
	Supp_Motor_Area	R	−3.1871	14	−6	66	79	0.00144
**Insula_G**		**R**						
	Fusiform	L	−3.7971	−40	−48	−8	182	0.00015
	Cingulum_Mid	L	−3.5536	−20	−56	16	31	0.00038
	Precentral	L	−3.3760	−44	−2	26	126	0.00074
	Caudate	R	4.9727	8	6	14	104	0.00001
**Insula_vIa**		**R**						
	Fusiform	L	−3.3560	−36	−36	−22	125	0.00079
	Caudate	R	−3.3863	6	4	−20	252	0.00071
	Temporal_Mid	R	−3.0368	48	−64	14	342	0.00239
	Precuneus	R	−4.5242	0	−42	24	634	0.00001
	Thalamus	R	3.7439	4	−24	18	410	0.00018
	Parietal_Inf	R	−3.4593	44	−50	38	79	0.00054
**Insula_dIa**		**R**						
	ParaHippocampal	R	−5.0413	26	−38	−8	286	0.00001
	Temporal_Inf	R	−3.3742	44	−64	2	239	0.00074
	Occipital_Mid	R	−3.3647	32	−88	16	270	0.00077
	Caudate	R	4.3188	14	−20	22	107	0.00002
	Cingulum_Mid	R	−4.0748	24	−64	24	219	0.00005
	Parietal_Sup	R	−4.8340	20	−72	62	330	0.00001
**Insula_vId/vIg**		**R**						
	Precuneus	R	−4.0771	0	−44	24	97	0.00007
	ParaHippocampal	R	−3.7133	30	−40	−4	100	0.00021
	Cuneus	R	−3.3512	22	−60	24	127	0.00081
	Cingulum_Mid	R	−3.2333	22	−16	36	34	0.00122
	Parietal_Inf	R	−3.5096	42	−58	60	286	0.00045
**Insula_dIg**		**R**						
	Temporal_Inf	L	−5.5472	−28	−28	−2	522	0.00001
	Lingual	R	−3.3785	2	−90	−14	104	0.00073
	Frontal_Sup_Medial	L	3.3894	−16	50	6	150	0.00070
	Supp_Motor_Area	R	−3.8541	6	0	24	123	0.00012
	Parietal_Inf	L	−3.7381	−34	−66	48	262	0.00019
**Insula_dId**		**R**						
	Precuneus	R	−4.9083	28	−38	−6	457	0.00001
	Frontal_Sup_Medial	R	3.9623	12	54	16	262	0.00007
	Precentral	R	−3.5556	34	−10	66	158	0.00038

*_Mid, _middle,_Sup, _superior,_Inf, _inferior, Lat, _lateral, L, left hemisphere, R, right hemisphere; t-values were two-sample t-test, t > 0 were FC enhanced, t <0 were FC decreased; p-values were two sample test at significance level in 0.05*.

**Figure 3:**
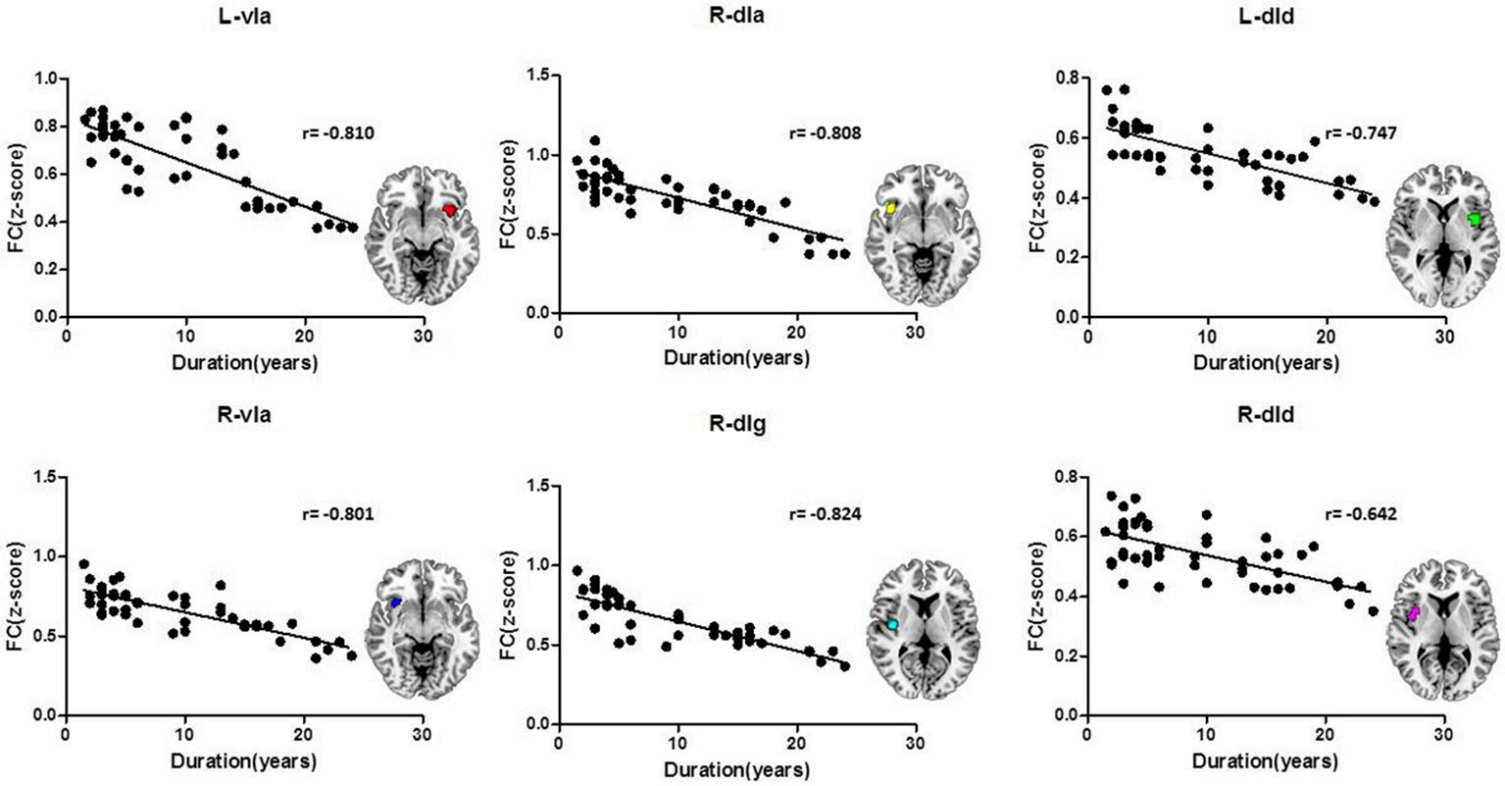
Correlation of the insular sub-region FC z-scores with the duration of MWoA. The insular sub-regions including L/R-vIa, L/R-dId, R-dIa, and R-dIg were negatively correlated with disease duration.

The FC z-scores were extracted using insular sub-regions as ROIs for the statistical analysis, and the results are shown in Table 4. The data indicated that the mean z-score of the insular sub-regions of the MWoA group was lower than that for the healthy control group. The *P*-values of the ANOVA of sub-regions such as the L/R-vIa, L/R-dId, R-dIa, and R-dIg were <0.05, and the *P*-values of the homogeneity test were >0.05. These were considered to be statistically significant results. These regions were more common in the right insula (four regions) than in the left insula (two regions). Furthermore, Pearson's test was applied to the statistically significant insular sub-regions, and the results are shown in Figure 3. The results indicated that these brain regions were negatively correlated with disease duration. These results suggest that the FC z-score of the insular sub-regions in MWoA patients gradually decreased with increasing disease duration.

**Table 4:** The results of FC z-scores were extracted using insular subregions as ROIs.

**Structure**	**Patients(*n* = 48)**	**Controls(*n* = 48)**	**Test^a^**		
	**z-score^b^**	**z-score^b^**	***P*^c^**	***F***	***P*^d^**
L-G	0.562 ± 0.113	0.581 ± 0.128	0.708	0.141	0.438
L-vIa	0.658 ± 0.154	0.735 ± 0.159	0.682	0.169	0.018^*^
L-dIa	0.760 ± 0.126	0.796 ± 0.147	0.673	0.180	0.220
L-vId/vIg	0.671 ± 0.122	0.704 ± 0.149	0.615	0.255	0.235
L-dIg	0.623 ± 0.129	0.644 ± 0.126	0.849	0.036	0.425
L-dId	0.552 ± 0.089	0.619 ± 0.107	0.515	0.427	0.001^*^
R-G	0.619 ± 0.119	0.629 ± 0.139	0.812	0.057	0.722
R-vIa	0.659 ± 0.134	0.752 ± 0.116	0.122	2.435	0.001^*^
R-dIa	0.695 ± 0.177	0.795 ± 0.150	0.096	2.835	0.004^*^
R-vId/vIg	0.656 ± 0.124	0.686 ± 0.140	0.859	0.032	0.425
R-dIg	0.657 ± 0.151	0.725 ± 0.145	0.371	0.808	0.025^*^
R-dId	0.543 ± 0.094	0.605 ± 0.121	0.134	2.290	0.007^*^

a Analysis of covariance and adjusted for age, gender and education years;

b The z-score were expressed as the mean ± SD;

c P values were test of homogeneity of variances;

d P values were analysis of covariance;

* *Significant p values after analysis of covariance (p < 0.05) and test of homogeneity of variances (P > 0.05)*.

## Discussion

The integrated experience of pain is composed of three processes, namely, sensation, emotion, and cognition (Schnitzler and Ploner, 2000). Therefore, the interpretation of a migraine can be derived from these three aspects.

The functions of the different insular gyri vary. Recently, brain function studies have reported that the anterior insula is associated with brain regions related to emotion regulation, primarily the temporal lobe and the limbic system (including the hippocampus, parahippocampal gyrus, amygdala, cingulate gyrus, thalamus and fusiform gyrus). In contrast, the posterior insula has been found to be primarily associated with the premotor area, sensorimotor area, supplementary motor area, medial/posterior cingulate gyrus, occipital lobe and parietal lobe (Cauda et al., 2011). In the insular subregion map used in this study, the vIa and vId/vIg have essentially the same position as the anterior insula in the abovementioned studies. Moreover, the G, dIa, dIg, and dId in the present study are equivalent to the posterior insula in previous studies. The present study found that the following FC-values were decreased in the MWoA patients: (i) between the L-vIa and the left thalamus; (ii) between the L-vIa and the left posterior cingulate gyrus; (iii) between the L-vId/vIg and the left superior temporal gyrus/the left fusiform gyrus; (iv) between the R-vIa and the right caudate nucleus, left middle temporal gyrus and left fusiform gyrus; and (v) between the R-vId/vIg and the center of the right cingulate gyrus, the right parahippocampal gyrus, the right fusiform gyrus, and the right cuneus. Organized brain functional activities occur in the resting state. The cuneus and cingulate are the brain regions with the greatest brain metabolic activity. The cingulate gyrus and cuneus continuously collect surrounding and internal information and allocate this information. The cuneus is involved in high-level cognitive functions related to episodic memory and self-relevant information processing and is associated with conscious short-term memory (Cabeza and Nyberg, 2000). The prefrontal lobe and medial hypothalamic nuclei and their projected limbic system constitute the affective component of pain, while the lateral hypothalamic nuclei and the corresponding cortical projection area and somatosensory area constitute the sensory pathway of pain (Schnitzler and Ploner, 2000). The anterior cingulate gyrus receives fiber projections from the midline thalamic nuclei and medial nuclear group of the thalamus and exhibits prominent functions in the affective component of pain (Jones et al., 1991). The FC of these brain regions was found to be decreased in the MWoA patients, which might be associated with the self-adaptation of the nervous system. Through the self-attenuation of the nervous system, emotional reactions to pain and the subjective feeling of pain are reduced as much as possible, and responses to sensory functions such as pain are also actively reduced. These data are consistent with previous findings, which demonstrated that the vIa and vId/vIg are primarily involved in the regulation of emotions in migraine patients. Leknes et al. found that stimulation of the hypothalamus and the medial/posterior cingulate gyrus resulted in an increased pain threshold (Leknes and Tracey, 2008). Thus, the decreased FC between the insula of the MWoA patients and the limbic system may be related to migraine. The limbic network participates in the development of migraine pain as well as in increasing the pain threshold. The FC between the vIa and vId/vIg of the MWoA patients and the limbic system decreased, which could be considered to indicate a weakened regulation of emotion.

This study also found that the FC between the L-dId and the right medial superior frontal gyrus was enhanced, whereas the FC between the L-dId and the right supplementary motor area, the central right cingulate gyrus, and the right inferior temporal gyrus was weakened. In addition, the FC between the R-dId and the right medial superior frontal gyrus increased, while the FC between the R-dId and the right precentral gyrus and the right precuneus decreased. The medial superior frontal gyrus and center of the cingulate gyrus are involved in emotional responses and the subjective sensation of pain, as well as pain-related memory, the attention response, and cognitive reaction (Bluhm et al., 2007). Additionally, this study found a weakened FC between the L-G and the right angular gyrus and the right precuneus as well as between the L-dIg and the left superior temporal gyrus, the left inferior temporal gyrus, the left precuneus, the central right cingulate gyrus, and the right inferior parietal angular gyrus. Moreover, the FC between the R-G and the left medial superior frontal gyrus, the left fusiform gyrus, and the center of the left cingulate gyrus was weakened. The FC between the R-dIg and the left medial superior frontal gyrus was enhanced, whereas the FC between the R-dIg and the right supplementary motor area, the right lingual gyrus, the left precuneus, and the left inferior parietal angular gyrus was weakened. The precuneus, the center of the cingulate gyrus, the inferior parietal angular gyrus, the medial superior frontal gyrus, and the temporal cortex are important components of the default network (Buckner et al., 2008). During the resting state, the default network is primarily associated with the higher functions of the human brain, such as memory and consciousness. In addition, the inferior parietal angular gyrus is primarily associated with the response to pain as well as with temperature and pressure sensation. The FC between the insula of the MWoA patients and these brain regions was weakened, an effect that may be related to the self-adaptation of the neural system. Self-attenuation of the nervous system can minimize emotional responses to pain and subjective sensations. In addition, this process can initially reduce the response to sensory stimuli, such as pain. A case-control study of chronic pain and healthy subjects demonstrated that the center of the cingulate gyrus contains a high density of opioid receptor binding sites (Jones et al., 1991), suggesting that this region participates in both the development and modulation of pain. Therefore, it can be concluded that the G, dIg, and dId are related to pain sensation and cognition in MWoA patients. The FC of the medial superior frontal gyrus is enhanced in MWoA patients, indicating that the perception of pain becomes more sensitive. The FC of other brain regions decreases, indicating a weakened regulation of emotion.

Notably, the brain regions that were determined to exhibit FC variation when using the bilateral vIa, dIa, vId/vIg, and R-dId as the seed points were in the ipsilateral hemisphere, whereas the brain regions exhibiting FC variations using the L-dId as the seed point were located in the contralateral hemisphere. We have no reasonable explanation for these results, although the findings may be related to the structure of the insula; this hypothesis requires further fiber tractography analysis in subsequent studies. We also found that the brain regions with FC variations using these seed points were more commonly on the right side of brain than on the left side, and the number of insular sub-regions with statistically significant FC changes was notably higher in the right insula than in the left insula, suggesting right insular dominance. These findings are in line with some previous studies (Ostrowsky et al., 2002). A previous study showed that von Economo neurons (VENs) develop earlier in right insular regions than they do in the left insula and that there are more VENs in right insular regions (Allman et al., 2010). Another group also suggested that the right insula plays a key role in self-awareness and social cognition (Critchley and Seth, 2012).

Studies have shown that migraine may be a progressive disease, and recurrent migraines increase the risk of subtle brain lesions that can lead to the functional impairment of certain brain regions (Schwedt and Dodick, 2009). The present study found that the FC z-scores of insular sub-regions in the MWoA group were lower than those of the control group. Moreover, the FC z-scores of some sub-regions were negatively correlated with disease duration, indicating that the z-score decreased with an extended disease course. Thus, these results suggest that MWoA is a progressive disease.

## Conclusion

Different insular sub-regions are functionally associated with different regions of the brain and therefore have different functions. The FC between the insular sub-regions and various brain regions was mostly reduced, while a small amount was increased; furthermore, the FC may be ipsilateral and have a right-side advantage in MWoA. The FC variations of the insular sub-regions were observed to be specific to MWoA. Because this study was cross-sectional and the sample size was relatively small, it is not clear whether the changes associated with the insular sub-regions in the MWoA patients are the results or the causes of headache. Therefore, to address this question, longitudinal neuroimaging studies that have larger sample sizes of MWoA patients and that use more advanced neuroimaging techniques are necessary.

## Ethics statement

This study was carried out in accordance with the recommendations of the Declaration of Helsinki. A written informed consent was obtained from all participants in this study. This study was approved by the Ethics Committee at the First Affiliated Hospital of the Third Military Medical University. And it was performed in accordance with the 1964 Declaration of Helsinki.

## Author contributions

ZY: Project conception and execution; writing of the first draft. YL: Participants recruitment and clinical data acquisition. LS: MRI data acquisitions. DL: Statistical analysis. XlH: MRI data analysis. XyH: Participants recruitment and clinical data acquisition. ZwZ: MRI data analysis. YW: Participants recruitment and MRI data acquisitions. QY: Participants recruitment and clinical data acquisition. JP: Participants recruitment and clinical data acquisition. ZhZ: Project conception, article review, and critique. HL: Project conception, article review, and critique.

### Conflict of interest statement

The authors declare that the research was conducted in the absence of any commercial or financial relationships that could be construed as a potential conflict of interest.
